# Macrolide Therapy in Patients with Sepsis or Septic Shock: A Systematic Review and Meta-Analysis

**DOI:** 10.3390/jcm14176254

**Published:** 2025-09-04

**Authors:** Kyeongdeok Kim, Jihye Lee

**Affiliations:** Division of Pulmonology and Allergy, Department of Internal Medicine, Soonchunhyang University Cheonan Hospital, Cheonan 31151, Republic of Korea; 121626@schmc.ac.kr

**Keywords:** macrolides, anti-inflammation, sepsis, septic shock

## Abstract

**Background**: Sepsis and septic shock are associated with markedly increased mortality. Some studies have suggested that macrolides improve outcomes independent of the antibiotic effects, possibly through immunomodulation. This study aimed to determine the effectiveness of macrolides in treating sepsis and septic shock. **Methods**: We searched electronic biomedical databases to investigate the effects of macrolide therapy versus non-macrolide therapy in patients with sepsis or septic shock. The primary outcome was overall mortality, which was also analyzed across subgroups according to the primary cause of sepsis and type of macrolides. Secondary outcomes consisted of the intensive care unit (ICU) length of stay (LOS) and hospital LOS. We calculated pooled risk ratio (RR) and mean difference (MD) with 95% confidence intervals (CIs) using a random-effects model, and statistical heterogeneity was assessed with the I^2^ statistic (I^2^). **Results**: We identified four randomized controlled trials (RCTs) (*n* = 1020) and seven observational studies (n = 2519). In RCTs, there was no significant difference in overall mortality between the macrolide and control groups (RR 1.08, 95% CI 0.92–1.27; I^2^ = 0%), whereas observational studies showed a statistically significant mortality reduction associated with macrolide therapy (RR 0.79, 95% CI 0.65–0.95; I^2^ = 49%). Subgroup analyses by macrolide type and sepsis cause in RCTs showed no significant differences. ICU and hospital LOS showed no significant differences between groups. **Conclusions**: Based on the current evidence from RCTs, adjunctive macrolide therapy may not provide a significant mortality benefit in patients with sepsis or septic shock. Further large-scale randomized trials are needed to better establish the role of macrolides in sepsis management.

## 1. Introduction

Sepsis and septic shock are significant global health challenges associated with high mortality and morbidity rates. Recent meta-analyses have reported 30-day mortality rates ranging from 24.4% to 34.7% and 90-day mortality rates from 32.2% to 38.5%, despite early identification and appropriate management strategies [1]. Sepsis is defined as life-threatening organ dysfunction caused by a dysregulated host response to infection [2]. The complex interaction between inflammatory and anti-inflammatory responses in sepsis presents significant diagnostic and therapeutic challenges owing to its unpredictable and highly individualized nature [3].

In patients with sepsis or septic shock, standard empiric antibiotic therapy generally consists of broad-spectrum β-lactam agents such as piperacillin–tazobactam, cefepime, with additional coverage directed by the suspected site of infection, prior antibiotic exposure, immune status, and local resistance patterns. In cases with a high likelihood of multidrug-resistant pathogens, including methicillin-resistant Staphylococcus aureus (MRSA) or resistant Gram-negative organisms, combination therapy may be considered [4,5]. These conventional antibiotics exert their effects primarily through bactericidal mechanisms, including inhibition of cell wall, protein, or DNA synthesis. In contrast, emerging evidence suggests that macrolide therapy may improve outcomes in critically ill patients independent of its antimicrobial activity. Beyond their role in inhibiting bacterial protein synthesis, macrolides modulate host immune responses by attenuating neutrophil activation and altering the production of pro- and anti-inflammatory cytokines, thereby providing potential immunomodulatory benefits in the context of sepsis and septic shock [6,7]. Macrolide therapy has been reported to suppress neutrophil chemotaxis, adhesion, and oxidative activity, while modulating both pro- and anti-inflammatory cytokine production [8]. Cytokines play a pivotal role in host defense during sepsis, with key mediators such as tumor necrosis factor-α (TNF-α), interleukin (IL)-1, IL-6, and IL-10 implicated in its pathophysiology [9]. Macrolides’ ability to modulate these cytokines may explain their potential therapeutic benefits [10]. This therapeutic effect was first identified in diffuse panbronchiolitis and subsequently reported in other chronic airway inflammatory diseases, including cystic fibrosis, asthma, and chronic obstructive pulmonary disease (COPD) [11].

A previous meta-analysis of 28 observational studies focused on critically ill patients with community-acquired pneumonia (CAP), rather than sepsis specifically [12]. Despite extensive research, the impact of macrolides on sepsis mortality rates remains uncertain. Additionally, the emergence of macrolide resistance and the risk of toxicities, such as QTc prolongation, have necessitated a reassessment of the risk–benefit profile of this treatment strategy [13]. Given these considerations, we conducted a systematic review and meta-analysis to assess the effect of macrolide therapy versus placebo or non-macrolide therapy on the overall mortality and secondary clinical outcomes in patients with sepsis.

## 2. Materials and Methods

### 2.1. Protocol and Registration

This systematic review and meta-analysis adhered to the guidelines outlined in The Cochrane Handbook for Systematic Reviews of Interventions and followed the Preferred Reporting Items for Systematic Reviews and Meta-Analyses (PRISMA) guidelines. The study protocol was prospectively registered in PROSPERO (CRD42023480028). As this study analyzed the existing literature rather than human subjects, it was exempt from Institutional Review Board approval. The PRISMA checklist was used to ensure comprehensive reporting.

### 2.2. Inclusion and Exclusion Criteria

We included studies that met the following criteria: (1) randomized controlled trials (RCTs) or observational studies; (2) studies involving adult patients aged 18 years or older diagnosed with sepsis or septic shock according to Sepsis-1, -2, or -3 consensus definitions or identified based on the International Classification of Diseases (ICD) codes; (3) studies using macrolides of any type as the intervention, with placebo or non-macrolide treatment as the control; (4) studies that reported mortality outcomes; and (5) studies published in English. The exclusion criteria were as follows: (1) case reports, case series with sample size < 20, editorials, and expert opinions; (2) duplicate studies; (3) conference abstracts or poster presentations without available full texts; (4) studies lacking a non-macrolide treatment control group or not reporting mortality; and (5) secondary analyses of the included studies.

### 2.3. Search Strategy

We conducted a comprehensive literature search with the assistance of an experienced librarian using major electronic biomedical databases: MEDLINE (via PubMed), Embase, Cochrane Central Register of Controlled Trials (CENTRAL), Ovid Medline, and Web of Science. The search strategy was developed using appropriate keywords and encompassed terms related to macrolide antibiotics, including ‘macrolides’ and specific agents (e.g., clarithromycin, azithromycin, erythromycin), as well as ‘sepsis’ and ‘septic shock’. Both free-text terms and Medical Subject Headings were used to ensure comprehensive coverage. The search included studies published up to December 2024. The complete search strategy for each database is provided in Supplementary Tables S1–S5.

### 2.4. Study Selection, Data Extraction

Two independent reviewers (K.K. and J.L.) assessed the titles and abstracts of identified studies for eligibility. Studies deemed potentially relevant by either reviewer during the initial evaluation underwent full-text review. Disagreements in the full-text review stage were resolved through discussion and consensus between the reviewers. Data were extracted from each study by two independent reviewers (K.K. and J.L.) using a standardized form. This form included study characteristics (authors, publication year, region, study design, cause of sepsis, and follow-up duration), patient demographics (age and sex), intervention details (type of macrolide, dosage, and duration of administration), and relevant outcomes. The primary outcome was the difference in mortality rates between the intervention and control groups. Secondary outcomes included intensive care unit (ICU) and hospital length of stay (LOS). When mortality was reported at multiple time points, long-term mortality was prioritized over short-term or in-hospital mortality. Subgroup analyses focusing on mortality were performed based on the cause of sepsis and the type of macrolide in RCTs.

### 2.5. Quality Assessment and Risk of Bias Evaluation

Two reviewers independently assessed the quality of included studies For RCTs, risk of bias was evaluated at the outcome level using the Cochrane Risk of Bias 2 (RoB 2) tool across five domains (randomization process; deviations from intended interventions; missing outcome data; measurement of the outcome; selection of the reported result), with judgments classified as low risk, some concerns, or high risk. For non-randomized studies of interventions, risk of bias was assessed using Risk Of Bias In Non-Randomized Studies of Interventions (ROBINS-I) across seven domains (confounding; selection of participants; classification of interventions; deviations from intended interventions; missing data; measurement of outcomes; selection of the reported result), with overall judgments of low, moderate, serious, or critical risk of bias. Additionally, the certainty of the evidence for RCTs was assessed using the Grading of Recommendations, Assessment, Development, and Evaluation (GRADE) approach. Discrepancies were resolved through discussions between the two reviewers until a consensus was reached.

### 2.6. Statistical Methods

Meta-analyses were performed using R software (version 4.5.1; R Foundation for Statistical Computing, Vienna, Austria) and Review Manager (RevMan, version 5.4; Cochrane Collaboration). For studies reporting LOS as median and interquartile range (IQR), means and standard deviations were estimated using the Luo method, which is appropriate for small sample sizes and skewed continuous data [14,15]. To reduce confounding by study design, RCTs and observational studies were analyzed separately. Random-effects models were used for all analyses considering the expected high heterogeneity in sepsis studies. Results were presented as forest plots. Dichotomous variables were summarized as risk ratios (RR) and continuous variables as mean differences (MD), each with corresponding 95% confidence intervals (CI). Statistical significance was defined as *p* < 0.05. Between-study heterogeneity was assessed using the I^2^ statistic as specified in the Cochrane Handbook for Systematic Reviews. Heterogeneity was interpreted as follows: I^2^ statistic (I^2^) = 0–40% (might not be important), 41–60% (moderate), 61–75% (substantial), 76–100% (considerable heterogeneity). When substantial heterogeneity (I^2^ > 50%) was observed, leave-one-out sensitivity analysis was conducted to assess the influence of individual studies on the pooled result. Subgroup analyses for 28/30-day mortality were performed in RCTs according to the cause of sepsis (pneumonia vs. all-cause sepsis) and type of macrolide (erythromycin, clarithromycin, or azithromycin). Publication bias assessment was not performed due to the limited number of studies (<10) in each analysis.

## 3. Results

### 3.1. Study Selection

**Figure 1:**
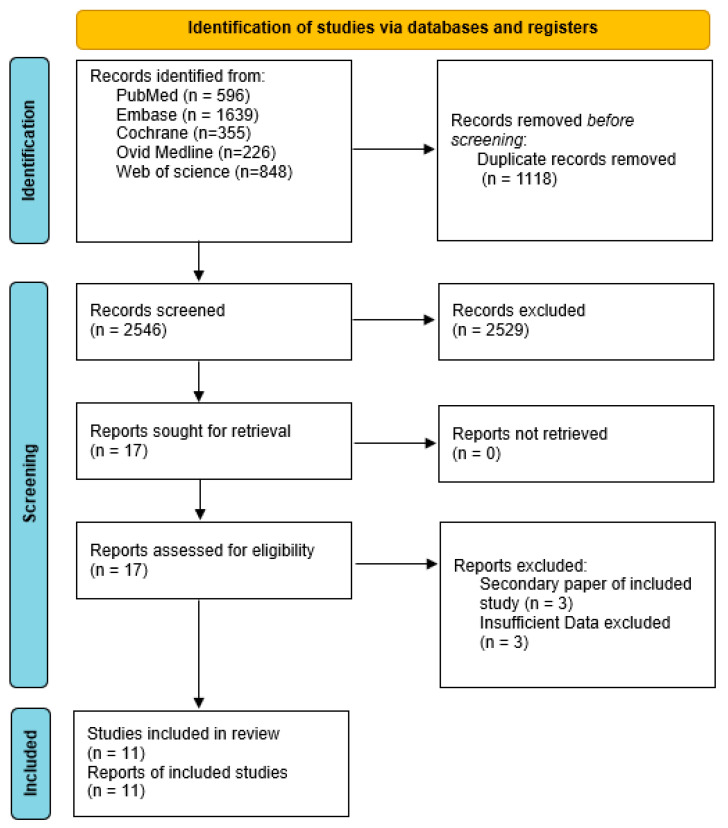
PRISMA flow diagram for study selection.

A total of 2546 studies were identified after removing duplicates. Initial screening of titles and abstracts led to the exclusion of 2529 studies that did not meet the inclusion criteria, leaving 17 studies for full-text review. Of these, six studies were further excluded because they were secondary publications (n = 3) or had insufficient data (n = 3). Consequently, a total of 11 studies [6,7,16,17,18,19,20,21,22,23,24], including 4 RCTs and 7 observational studies, met the inclusion criteria and were included in this meta-analysis. The study selection process is summarized in the PRISMA flow diagram (Figure 1).

### 3.2. Study and Patient’s Characteristics

Table 1 summarizes the characteristics of the included studies and the patients included in the meta-analysis. All studies were published between April 2008 and June 2024, focusing on patients with sepsis or septic shock. In terms of origin, four studies [6,17,20,23] were from the USA, five [16,18,19,21,22] from Europe (France, Greece, Belgium, and the Netherlands), one [7] from Japan, and one [24] from Tunisia. Of the 11 studies, 4 were RCTs [18,19,21,24], 2 were prospective cohort studies [16,22], and 5 were retrospective cohort studies [6,7,17,20,23]. The total sample size across these studies was 3539 patients, of whom 1325 received macrolide treatment and 2214 received placebo or no macrolide therapy. The mean age of participants ranged from 55.2 to 75 years and the proportion of males ranged from 45% to 99%. Regarding the type of macrolide, four studies [6,7,17,20] focused on patients treated with azithromycin, three on clarithromycin [18,19,21], and two on erythromycin [22,24], while two studies [16,23] did not specify the type of macrolide used.

### 3.3. Primary Outcome

**Figure 2:**
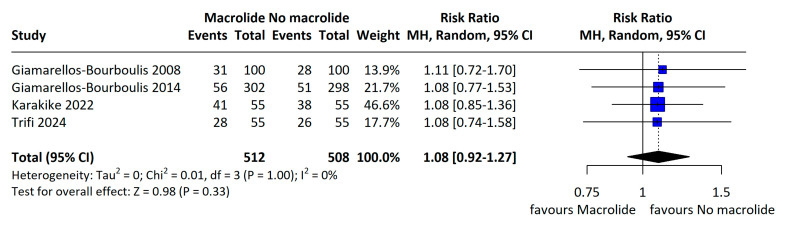
Primary outcome: mortality in RCT. A random-effects forest plot of mortality from RCTs. When multiple time points were available, the longest follow-up was prioritized. Giamarellos-Bourboulis 2008 [8] and 2014 [18], Trifi 2024 [24] studies used 28/30-day mortality, Karakike 2022 [21] study used 90-day mortality.

**Figure 3:**
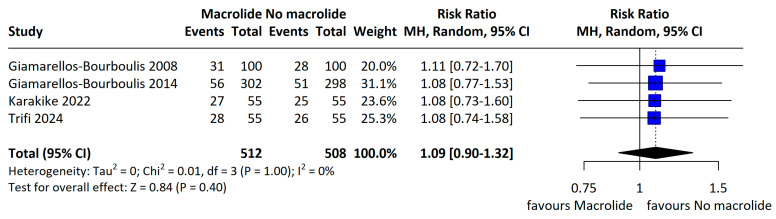
Primary outcome: 28/30 day mortality in RCT (adapted from [8,19,21,24]).

**Figure 4:**
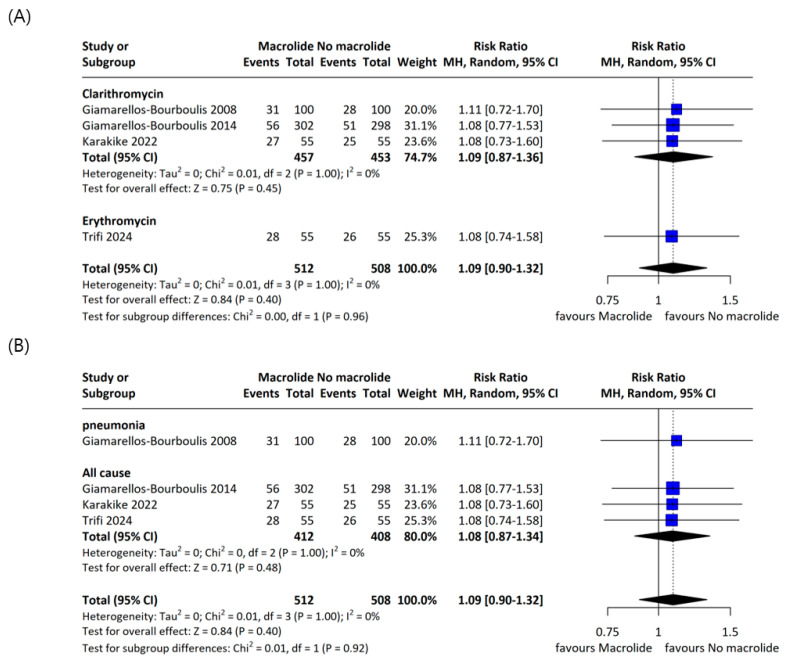
Subgroup analysis of 28-day mortality in RCTs by macrolide type and sepsis cause. Forest plots showing subgroup analyses of 28-day mortality by (**A**) macrolide type (clarithromycin versus erythromycin) (adapted from [8,19,21,24])and (**B**) cause of sepsis (pneumonia-associated sepsis versus all-cause sepsis) (adapted from [8,19,21,24]).

The individual study results included in the meta-analysis are presented in Table 2. Across four RCTs enrolling 1020 patients, the pooled all-cause mortality was 30.5% in the macrolide group and 28.2% in the non-macrolide group; although mortality tended to be higher with macrolides, the difference was not statistically significant (RR 1.08, 95% CI 0.92–1.27; I^2^ = 0%; Figure 2). When restricted to 28-day mortality, rates were 27.7% and 25.9% in the macrolide and non-macrolide groups, respectively, again with no significant difference (RR 1.09, 95% CI 0.90–1.32; I^2^ = 0%; Figure 3). Subgroup analyses within the RCTs by macrolide agent and by sepsis etiology likewise showed no significant differences in 28-day mortality (Figure 4A,B).

**Figure 5:**
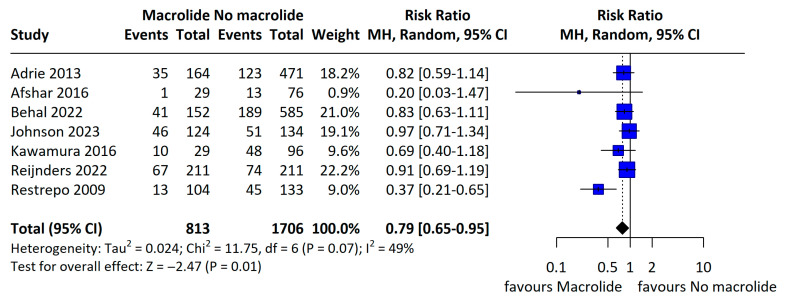
Primary outcome: mortality in observational studies. A random-effects forest plot of mortality from observational studies only. When multiple time points were available, the longest follow-up was prioritized. Afshar 2016 study [6] used 28-day mortality, Adrie 2013 [16], and Kawamura 2016 [7] studies used 60-day mortality, Reijnders 2022 [22], Restrepo 2009 [23] studies used 90-day mortality, and Behal 2022 [17], Johnson 2023 [20] studies in-hospital mortality used.

In contrast, when we analyzed observational studies only, overall mortality was 26.2% in the macrolide group versus 30.1% in the non-macrolide group, indicating a statistically significant reduction associated with macrolide therapy (RR 0.79, 95% CI 0.65–0.95; I^2^ = 49%; Figure 5).

### 3.4. Secondary Outcome

**Figure 6:**
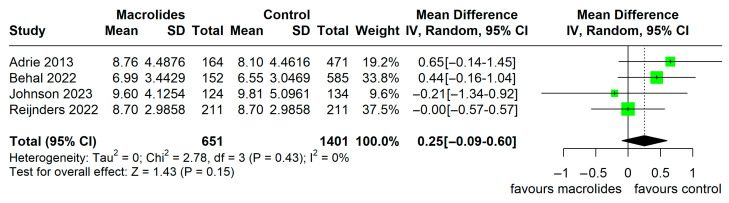
ICU LOS in observational studies (adapted from [16,17,20,22]).

ICU LOS was reported in four observational studies and two RCTs. Meta-analysis of observational studies revealed no significant difference in ICU LOS between the macrolide treatment group and the control group (MD 0.25 days, 95% CI −0.09 to 0.60, I^2^ = 0%, Figure 6). For RCTs, ICU LOS was reported in only two studies with conflicting directions of effect and considerable heterogeneity (I^2^ = 94%), precluding meaningful meta-analysis. Individual study results are presented descriptively in Table 2.

**Figure 7:**
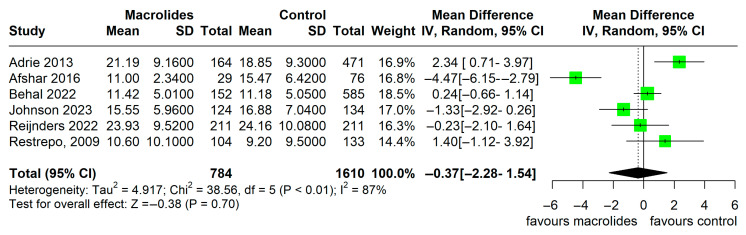
Hospital LOS in observational studies (adapted from [6,16,17,20,22,23]).

Hospital LOS was reported in six observational studies, showing no significant difference between groups (MD −0.37 days, 95% CI −2.28 to 1.54, I^2^ = 87%, Figure 7). Due to the substantial heterogeneity (I^2^ = 87%), we conducted a leave-one-out sensitivity analysis. When the Afshar 2016 [6] study was excluded, the pooled mean difference changed from −0.37 days to 0.36 days (Δ0.73 days); however, none of the analyses reached statistical significance.

### 3.5. Quality of Included Studies and Certainty of Evidence

The risk of bias for four RCTs was assessed using Cochrane RoB 2 tool; the overall judgments were low in two studies and some concerns in two studies (Figure S1). The seven observational studies were evaluated with ROBINS-I, with overall judgments of low in two studies, moderate in three, and serious in two (Table S5). The certainty of evidence for overall mortality was rated as low due to indirectness and imprecision (Table S5).

## 4. Discussion

This systematic review and meta-analysis involving 11 studies, including four RCTs and seven observational studies, evaluated the effectiveness of macrolide therapy in patients with sepsis or septic shock. In RCT analysis, macrolide therapy did not significantly affect overall mortality (RR 1.08, 95% CI 0.92–1.27; I^2^ = 0%) or 28-day mortality (RR 1.09, 95% CI 0.90–1.32; I^2^ = 0%). Subgroup analyses by macrolide type and sepsis etiology in RCTs also showed no significant differences. In contrast, observational studies showed a statistically significant mortality reduction associated with macrolide therapy (RR 0.79, 95% CI 0.65–0.95; I^2^ = 49%). There were no significant differences between groups in ICU or hospital LOS.

Despite extensive research on macrolides’ role in sepsis, their therapeutic impact remains controversial. One cohort study reported increased ICU-free days in severe sepsis patients [6] treated with azithromycin, whereas another study reported a reduced risk of death from septic shock and multiple organ dysfunction in patients with sepsis and ventilator-associated pneumonia treated with clarithromycin [25]. In contrast, one RCT evaluating clarithromycin versus placebo in granuloma-negative sepsis showed no difference in 28-day mortality [18]. A large prospective observational study in patient with acute respiratory distress syndrome (ARDS) demonstrated an association between macrolide therapy and reduced mortality (Odds ratio 0.64, 95% CI 0.43–0.96, *p* = 0.03) [26]. However, in the absence of direct mechanistic data or subgroup evidence from RCTs specifically targeting pulmonary infections, further research is warranted.

Macrolides exert their antimicrobial effect by binding to the 50S ribosomal subunit of bacteria, thereby inhibiting protein synthesis, suppressing toxin production, and limiting the growth of both extracellular and intracellular pathogens relevant to sepsis [27]. However, beyond their antimicrobial activity we propose that the survival benefit of macrolide therapy in sepsis may be attributed more to its anti-inflammatory and immunomodulatory effects than its antimicrobial activity, as observed in animal models [28,29,30]. To control for the potential survival benefits of antibiotics, two RCTs conducted by Giamarellos-Bourboulis et al. included only patients with sepsis caused by macrolide-resistant pathogens [18,19]. The immunomodulatory effects of macrolides involve targeting neutrophils by reducing their adhesion and promoting apoptosis, thus contributing to their anti-inflammatory profile [31]. Additionally, macrolides inhibit the phosphorylation of extracellular signal-regulated kinase 1/2 and the activation of nuclear factor kappa B, crucial elements in intracellular signaling. These leads to reduced levels of pro-inflammatory cytokines such as TNF-α, IL-1β, IL-8, and IL-6, promoting a more anti-inflammatory state [32]. Furthermore, macrolide therapy has been shown to lower the serum IL-10 to TNF-α ratio and to promote the production of TNF-α, IL-6, and soluble triggering receptor expressed on myeloid cells-1 (sTREM-1) from activated monocytes, indicating a potential role in re-establishing the equilibrium between pro- and anti-inflammatory mediators in sepsis [25]. Collectively, these mechanisms may explain the potential immunomodulatory effects of macrolides, but their clinical efficacy requires further clarification through additional research.

The combination of macrolides with standard sepsis antibiotics such as β-lactams, carbapenems, and aminoglycosides is generally safe, as these agents have minimal metabolic interactions, though cumulative nephrotoxicity or ototoxicity requires monitoring [33]. In contrast, co-administration with fluoroquinolones raises concern for QT interval prolongation and potential arrhythmias, necessitating electrocardiographic monitoring in high-risk patients [34]. Overall, macrolide combination therapy in sepsis is considered safe when clinicians account for drug-specific interaction profiles and patient comorbidities.

In our analysis of critically ill patients with sepsis, RCTs did not demonstrate mortality benefit with adjunctive macrolide therapy. Several factors may explain the discrepancy between RCT and observational study findings. As noted by Trifi et al., fundamental differences exist between sepsis and chronic respiratory diseases where macrolides have proven beneficial. In sepsis, the profound imbalance between pro- and anti-inflammatory responses, coupled with capillary leak and vasoplegia, may alter drug pharmacokinetics and distribution [24]. Additionally, variability in the specific agents used, sources of infection, dosing regimens, and duration of administration across studies may have influenced mortality outcomes and contributed to the observed heterogeneity between study types.

This study has several limitations. First, the limited number of included RCTs (four studies) may result in insufficient statistical power, and observational studies are inherently susceptible to selection bias and unmeasured confounding variables. Despite conducting separate analyses by study design, these methodological limitations may influence result interpretation, and well-designed large-scale randomized controlled trials are needed to provide more robust evidence. Second, despite using random-effects models, substantial heterogeneity was observed in some outcomes. Particularly high heterogeneity (I^2^ = 87%) was found in hospital LOS, necessitating leave-one-out sensitivity analysis. This heterogeneity likely stemmed from clinical and methodological diversity, including differences in patient characteristics, sepsis severity, and treatment protocols among the included studies. Third, the sepsis cohort was inherently heterogeneous, making it difficult to control for variability in sepsis etiology and severity. Improved stratification methods for sepsis patients are needed to gain a more precise understanding of this diverse patient population. Finally, formal assessment of publication bias could not be performed due to the limited number of studies. Future research should focus on determining optimal dosing, treatment duration, and timing of administration, comparing differential effects among macrolide types (azithromycin, clarithromycin, erythromycin), and identifying patient subgroups most likely to benefit from treatment (such as pneumonia-associated sepsis or specific inflammatory phenotypes). Additionally, systematic evaluation of safety profiles and drug interactions should be conducted concurrently.

Despite these limitations, this study has several strengths. First, we included a total of 11 studies (including four RCTs) with 3539 adults with sepsis, providing a comprehensive analysis of currently available evidence. To our knowledge, this is the first systematic review and meta-analysis to separately analyze RCTs and observational studies investigating macrolide therapy in sepsis patients. Second, we performed subgroup analyses based on sepsis etiology and macrolide type in RCTs to evaluate the robustness of our findings. Third, we conducted sensitivity analysis to address heterogeneity and performed separate analyses by study design to minimize confounding. Finally, the certainty of evidence for RCTs was assessed using the GRADE approach.

In conclusion, adjunctive macrolide therapy did not significantly reduce overall mortality in patients with sepsis or septic shock based on evidence from randomized controlled trials. Although observational studies showed potential benefit, this may be attributable to residual confounding variables. Therefore, current evidence is insufficient to support routine use in clinical practice, and well-designed large-scale randomized controlled trials are needed.

## Figures and Tables

**Table 1:** Study and patient characteristics of the included studies.

Study, Year	Study Design	Site	Total n (Macrolide/Control)	Male, n	Age, Mean ± SD or Median (IQR), Years	Type of Drug	Dose of Macrolide (mg)	Cause of Sepsis	Follow-Up Duration
Adrie, 2013 [16]	PC	12 ICUs, France	635 (164/471)	411	62 (46–76)	NR	NR	Pneumonia	60 days
Afshar, 2016 [6]	RC	Single center, USA	105 (29/76)	63	55.2 ± 16.4	Azithromycin	NR	All cause	NR
Behal, 2022 [17]	RC	Single center, USA	737 (152/585)	411	61 (50–70)/ 56 (45–66)	Azithromycin	NR	All cause	NR
Giamarellos-Bourboulis, 2008 [8]	RCT	Multicenter, Greece	200 (100/100)	147	58.4 ± 19.1	Clarithromycin	1000	Pneumonia	28 days
Giamarellos-Bourboulis, 2014 [18]	RCT	Multicenter, Greece	600 (302/298)	270	66.9 ± 19.6	Clarithromycin	1000	All cause	28 days
Johnson, 2023 [20]	RC	3 ICUs, USA	258 (124/134)	147	62.2 ± 12.8	Azithromycin	NR	All cause	NR
Karakike, 2022 [21]	RCT	Multicenter, Greece and Belgium	110 (55/55)	72	74 (62–80)	Clarithromycin	1000	All cause	90 days
Kawamura, 2016 [7]	RC	Single center, Japan	125 (29/96)	85	75 (66–82)	Azithromycin	NR	All cause	60 days
Reijnders, 2022 [22]	PC	2 ICUs, The Netherlands	705 (235/470)	447	60.9 ± 14.8	Erythromycin	120–250 mg (up to 600 mg/d)	All cause	90 days
Restrepo, 2009 [23]	RC	2 ICUs, USA	237 (104/133)	189	61.3 ± 16.7	NR	NR	Pneumonia	90 days
Trifi, 2024 [24]	RCT	Single center, Tunisia	110 (55/55)	70	63 (51–78)/64 (54–77)	Erythromycin	3000	All cause	28 days

Abbreviations: ICU: intensive care unit, IQR: interquartile range, NR: not reported, RCT: randomized controlled trials, PC: prospective cohort, RC: retrospective cohort, SD: standard deviation.

**Table 2:** Outcomes of the included studies in the meta-analysis.

Study, Year	Overall Mortality, n (Macrolide/Control)	28/30-Day Mortality, n (Macrolide/ Control)	60-Day Mortality, n (Macrolide/ Control)	90-Day Mortality, n (Macrolide/ Control)	ICU LOS, Mean ± SD or Median (IQR), Days (Macrolide/ Control)	Hospital LOS, Mean ± SD or Median (IQR), Days (Macrolide/ Control)
Adrie, 2013 [16]	35/123	NR	35/123	NR	7 (3.5–15.5)/6 (3–15)	17.5 (10.5–35)/15 (8–33)
Afshar, 2016 [6]	1/13	1/13	NR	NR	NR	11 (8–14)/13 (8–25)
Behal, 2022 [17]	41/189	NR	NR	NR	5.8 (2.9–12.1)/5.5 (2.9–11.1)	10.3 (5.2–18.6)/9.5 (5.1–18.7)
Giamarellos-Bourboulis, 2008 [8]	21/24	21/24	NR	NR	23.4 (±7.1)/21.6 (±8.22)	NR
Giamarellos-Bourboulis, 2014 [18]	56/51	56/51	NR	NR	NR	NR
Johnson, 2023 [20]	46/51	NR	NR	NR	8.4 (4.6–15.6)/8.4 (3.6–17.2)	13.9 (8.3–24.2)/15.4 (8.1–26.9)
Karakike, 2022 [21]	41/38	27/25	NR	41/38	NR	NR
Kawamura, 2016 [7]	10/48	NR	10/48	NR	NR	NR
Reijnders, 2022 [22]	67/74	54/56	NR	67/74	8 (5–13)/8 (5–13)	22 (12–37.5)/21 (12–39)
Restrepo, 2009 [23]	13/45	11/37	NR	13/45	NR	10.6 (±10.1)/9.2 (±9.5)
Trifi, 2024 [24]	28/26	28/26	NR	NR	15 (12–18)/18 (11–28)	NR

Abbreviations: ICU: intensive care unit, LOS: length of stay, NR: not reported, SD: standard deviation, IQR: interquartile range.

## Data Availability

All data used in this study is provided within the article.

## Supplementary Materials

The following supporting information can be downloaded at https://www.mdpi.com/article/10.3390/jcm14176254/s1, Figures S1–S7: Assessment of risk of bias; Table S1: Search strategy for PubMed; Table S2: Search strategy for Ovid Medline; Table S3: Search strategy for Cochrane Library; Table S4: Search strategy for the Web of science; Table S5: Quality assessment of observational studies using the nine-star Newcastle-Ottawa Scale.
