# Supplementary material for: Macrolide Therapy in Patients with Sepsis or Septic Shock: A Systematic Review and Meta-Analysis
Source: J Clin Med. 2025 Sep 4;14(17):6254. doi: 10.3390/jcm14176254 (PMC12429649; doi:10.3390/jcm14176254)
Supplement: Supplementary file 1 [file jcm-14-06254-s001.zip › jcm-3819136-supplementary.pdf]

Macrolide in patients with sepsis or septic shock: A systematic review and meta-analysis

Supplementary Figure S1. Primary outcome: 28/30-day mortality

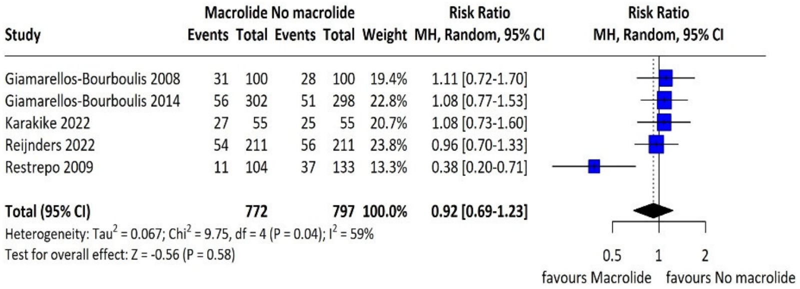

Supplementary Figure S2. Primary outcome : 90-day mortality

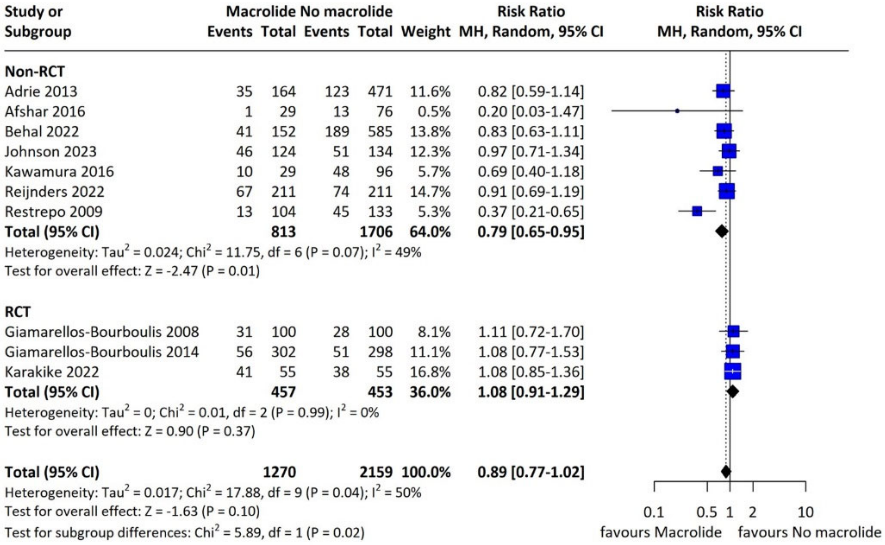

Supplementary Figure S3. Primary outcome : Hospital mortality

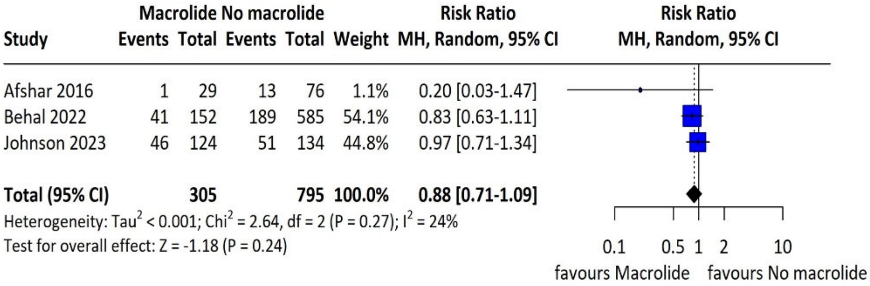

Supplementary Figure S4. Subgroup analysis by macrolide type

Study or Subgroup	Macrolide Events	Macrolide Total	No macrolide Events	No macrolide Total	Weight	Risk Ratio MH, Random, 95% CI
**Macrolide**						
Adrie 2013	35	164	123	471	11.6%	0.82 [0.59-1.14]
Restrepo 2009	13	104	45	133	5.3%	0.37 [0.21-0.65]
**Total (95% CI)**		**268**		**604**	**17.0%**	**0.57 [0.26-1.23]**
Heterogeneity: $Tau^2 = 0.260$; $Chi^2 = 5.7$, df = 1 (P = 0.02); $I^2 = 82\%$						
Test for overall effect: Z = -1.43 (P = 0.15)						
**Azithromycin**						
Afshar 2016	1	29	13	76	0.5%	0.20 [0.03-1.47]
Behal 2022	41	152	189	585	13.8%	0.83 [0.63-1.11]
Johnson 2023	46	124	51	134	12.3%	0.97 [0.71-1.34]
Kawamura 2016	10	29	48	96	5.7%	0.69 [0.40-1.18]
**Total (95% CI)**		**334**		**891**	**32.3%**	**0.85 [0.70-1.04]**
Heterogeneity: $Tau^2 = < 0.001$; $Chi^2 = 3.33$, df = 3 (P = 0.34); $I^2 = 10\%$						
Test for overall effect: Z = -1.59 (P = 0.11)						
**Clarithromycin**						
Giamarellos-Bourboulis 2008	31	100	28	100	8.1%	1.11 [0.72-1.70]
Giamarellos-Bourboulis 2014	56	302	51	298	11.1%	1.08 [0.77-1.53]
Karakike 2022	41	55	38	55	16.8%	1.08 [0.85-1.36]
**Total (95% CI)**		**457**		**453**	**36.0%**	**1.08 [0.91-1.29]**
Heterogeneity: $Tau^2 = 0$; $Chi^2 = 0.01$, df = 2 (P = 0.99); $I^2 = 0\%$						
Test for overall effect: Z = 0.90 (P = 0.37)						
**Erythromycin**						
Reijnders 2022	67	211	74	211	14.7%	0.91 [0.69-1.19]
**Total (95% CI)**		**1270**		**2159**	**100.0%**	**0.89 [0.77-1.02]**
Heterogeneity: $Tau^2 = 0.017$; $Chi^2 = 17.88$, df = 9 (P = 0.04); $I^2 = 50\%$						
Test for overall effect: Z = -1.63 (P = 0.10)						
Test for subgroup differences: $Chi^2 = 5.14$, df = 3 (P = 0.16)						

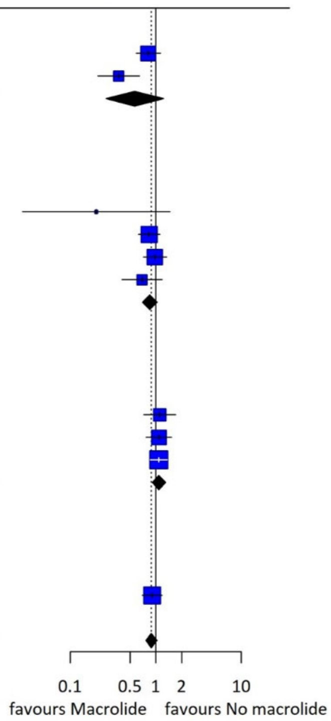

Supplementary Figure S5. Subgroup analysis by cause of sepsis

Study or Subgroup	Macrolide Events	Macrolide Total	No macrolide Events	No macrolide Total	Weight	Risk Ratio MH, Random, 95% CI
**pneumonia**						
Adrie 2013	35	164	123	471	11.6%	0.82 [0.59-1.14]
Giamarellos-Bourboulis 2008	31	100	28	100	8.1%	1.11 [0.72-1.70]
Restrepo 2009	13	104	45	133	5.3%	0.37 [0.21-0.65]
**Total (95% CI)**		**368**		**704**	**25.1%**	**0.71 [0.39-1.31]**
Heterogeneity: $Tau^2 = 0.236$; $Chi^2 = 9.44$, df = 2 (P < 0.01); $I^2 = 79\%$						
Test for overall effect: Z = -1.09 (P = 0.27)						
**All cause**						
Afshar 2016	1	29	13	76	0.5%	0.20 [0.03-1.47]
Behal 2022	41	152	189	585	13.8%	0.83 [0.63-1.11]
Giamarellos-Bourboulis 2014	56	302	51	298	11.1%	1.08 [0.77-1.53]
Johnson 2023	46	124	51	134	12.3%	0.97 [0.71-1.34]
Karakike 2022	41	55	38	55	16.8%	1.08 [0.85-1.36]
Kawamura 2016	10	29	48	96	5.7%	0.69 [0.40-1.18]
Reijnders 2022	67	211	74	211	14.7%	0.91 [0.69-1.19]
**Total (95% CI)**		**902**		**1455**	**74.9%**	**0.95 [0.84-1.07]**
Heterogeneity: $Tau^2 = < 0.001$; $Chi^2 = 6.3$, df = 6 (P = 0.39); $I^2 = 5\%$						
Test for overall effect: Z = -0.83 (P = 0.41)						
**Total (95% CI)**		**1270**		**2159**	**100.0%**	**0.89 [0.77-1.02]**
Heterogeneity: $Tau^2 = 0.017$; $Chi^2 = 17.88$, df = 9 (P = 0.04); $I^2 = 50\%$						
Test for overall effect: Z = -1.63 (P = 0.10)						
Test for subgroup differences: $Chi^2 = 0.82$, df = 1 (P = 0.36)						

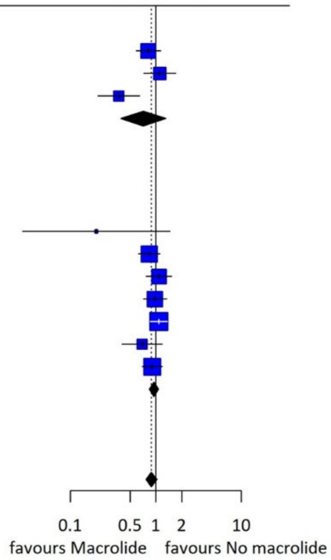

Supplementary Figure S6. Assessment of risk of bias

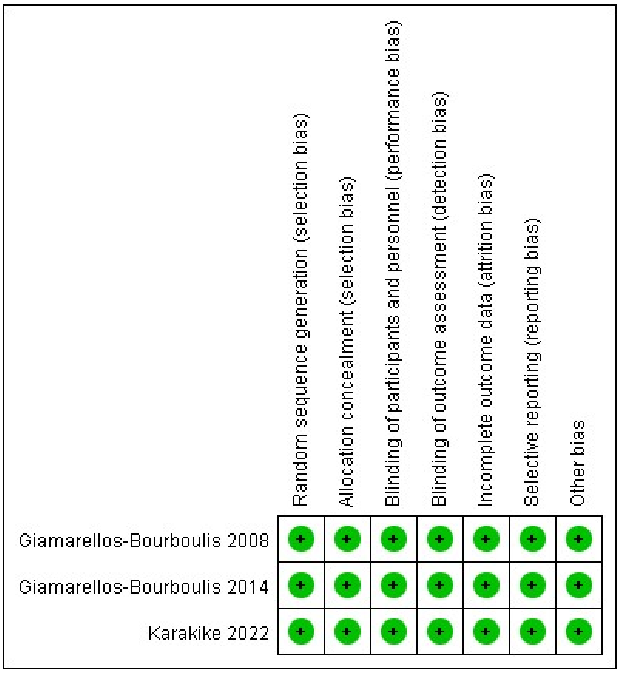

Supplementary Figure S7. Funnel plot for studies reporting overall mortality

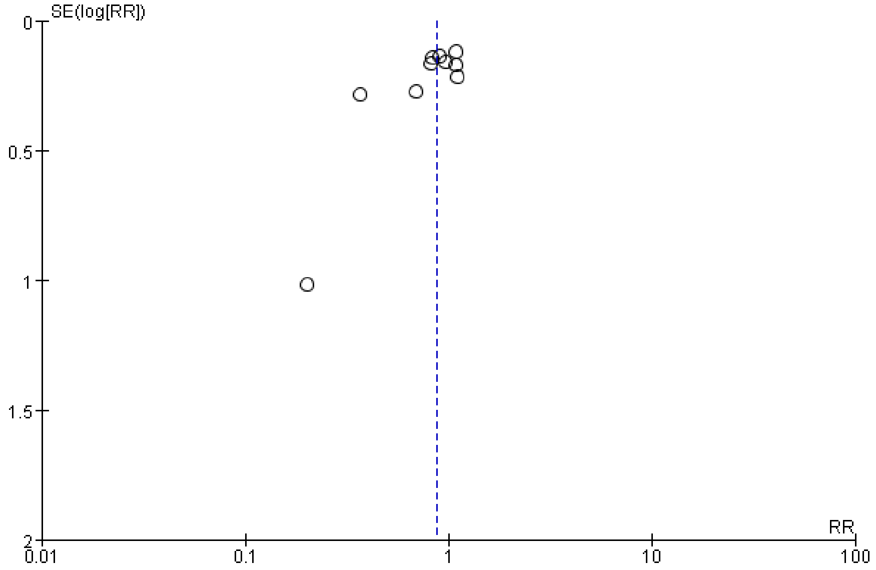

Supplementary Table S1 Search strategy for PubMed

No.	Search Query	Results
#1	Shock, Septic[mh] OR Sepsis[mh] OR Septic Shock[tiab] OR Shock, Septic[tiab] OR Shock, Endotoxi*[tiab] OR Endotoxin Shock*[tiab] OR Endotoxic Shock*[tiab] OR Sepsis[tiab] OR Bloodstream Infection*[tiab] OR Infection, Bloodstream[tiab] OR Pyemia*[tiab] OR Pyohemia*[tiab] OR Pyaemia*[tiab] OR Septicemia*[tiab] OR Poisoning, Blood[tiab] OR Poisonings, Blood[tiab] OR Blood Poisoning*[tiab] OR Severe Sepsis[tiab] OR Sepsis, Severe[tiab]	265,530
#2	Macrolides[mh] OR Erythromycin[mh] OR Azithromycin[mh] OR Clarithromycin[mh] OR Erythromycin Estolate[mh] OR Erythromycin Ethylsuccinate[mh] OR Ketolides[mh] OR Roxithromycin[mh] OR macrolide 2'-kinase[Supplementary Concept] OR YC-17 macrolide[Supplementary Concept] OR mirabilin[Supplementary Concept] OR RAD macrolide[Supplementary Concept] OR macrolide glycosyltransferase[Supplementary Concept] OR erythromycin G[Supplementary Concept] OR erythromycin 2'-acetate[Supplementary Concept] OR "(N-methyl) erythromycin"[Supplementary Concept] OR erythromycin A isoxazoline[Supplementary Concept] OR erythromycin E[Supplementary Concept] OR erythromycin esterase[Supplementary Concept] OR erythromycin propionate[Supplementary Concept] OR 14-R-hydroxy-clarithromycin[Supplementary Concept] OR O-demethyl-	146,405

	roxithromycin[Supplementary Concept] OR Macrolide*[tiab] OR Erythromycin*[tiab] OR T-Stat[tiab] OR Erymax[tiab] OR Erycette[tiab] OR Ilotycin[tiab] OR Azythromycin[tiab] OR Sumamed[tiab] OR CP-62993[tiab] OR Zithromax[tiab] OR Azitrocin[tiab] OR Azadose[tiab] OR Ultreon[tiab] OR Zitromax[tiab] OR Clarithromycin[tiab] OR 6-O-Methylerythromycin[tiab] OR TE-031[tiab] OR A-56268[tiab] OR Biaxin[tiab] OR Erythromycin Estolate[tiab] OR Erythromycin*[tiab] OR Ilosone[tiab] OR Erythromycin Ethylsuccinate[tiab] OR Wyamycin E[tiab] OR Monomycin[tiab] OR E-Mycin E[tiab] OR Erythroped[tiab] OR Ketolides[tiab] OR Roxithromycin[tiab] OR Rulide[tiab] OR Rulid[tiab] OR Roxi*[tiab] OR RU-28965[tiab] OR RU-965[tiab]	
#3	#1 AND #2	2,959
#4	#3 NOT (casereports[Filter] OR review[Filter]) AND (english[Filter]) AND (adult[Filter])	577
Total		577

Supplementary Table S2 Search strategy for Ovid Medline

No.	Search Query	Results

#1	(Shock, Septic or Sepsis).sh. or (Septic Shock or Shock, Septic or Shock, Endotoxi* or Endotoxin Shock* or Endotoxic Shock* or Sepsis or Bloodstream Infection* or Infection, Bloodstream or Pyemia* or Pyohemia* or Pyaemia* or Septicemia* or Poisoning, Blood or Poisonings, Blood or Blood Poisoning* or Severe Sepsis or Sepsis, Severe).ab,ti.	193,998
#2	(Macrolides or Erythromycin or Azithromycin or Clarithromycin or Erythromycin Estolate or Erythromycin Ethylsuccinate or Ketolides or Roxithromycin).sh. or (Macrolide* or Erythromycin* or T-Stat or Erymax or Erycette or Ilotycin or Azythromycin or Sumamed or CP-62993 or Zithromax or Azitrocin or Azadose or Ultreon or Zitromax or Clarithromycin or 6-O-Methylerythromycin or TE-031 or A-56268 or Biaxin or Erythromycin Estolate or Erythromycin* or Ilosone or Erythromycin Ethylsuccinate or Wyamycin E or Monomycin or E-Mycin E or Erythroped or Ketolides or Roxithromycin or Rulide or Rulid or Roxi* or RU-28965 or RU-965).ab,ti.	66,081
#3	#1 AND #2	1,030
#4	#3 NOT (casereports[Filter] OR review[Filter]) AND (english[Filter]) AND (adult[Filter])	176
Total		176

Supplementary Table S3 Search strategy for Cochrane Library

No.	Search Query	Results
#1	MeSH descriptor: [Shock, Septic] explode all trees or MeSH descriptor: [Sepsis] explode all trees or (Septic Shock or Shock, Septic or Shock, Endotoxi* or Endotoxin Shock* or Endotoxic Shock* or Sepsis or Bloodstream Infection* or Infection, Bloodstream or Pyemia* or Pyohemia* or Pyaemia* or Septicemia* or Poisoning, Blood or Poisonings, Blood or Blood Poisoning* or Severe Sepsis or Sepsis, Severe):ti,ab,kw	19,763
#2	MeSH descriptor: [Macrolides] explode all trees or MeSH descriptor: [Erythromycin] explode all trees or MeSH descriptor: [Azithromycin] explode all trees or MeSH descriptor: [Clarithromycin] explode all trees or MeSH descriptor: [Erythromycin Estolate] explode all trees or MeSH descriptor: [Erythromycin Ethylsuccinate] explode all trees or MeSH descriptor: [Ketolides] explode all trees or MeSH descriptor: [Roxithromycin] explode all trees or (Macrolide* or Erythromycin* or T-Stat or Erymax or Erycette or Ilotycin or Azythromycin or Sumamed or CP-62993 or Zithromax or Azitrocin or Azadose or Ultreon or Zitromax or Clarithromycin or "6-O-Methylerythromycin" or TE-031 or A-56268 or Biaxin or Erythromycin Estolate or Erythromycin* or Ilosone or Erythromycin Ethylsuccinate or	14,549

	Wyamycin E or Monomycin or E-Mycin E or Erythroped or Ketolides or Roxithromycin or Rulide or Rulid or Roxi* or RU-28965 or RU-965):ti,ab,kw	
#3	#1 AND #2	319
Total		319

Supplementary Table S4 Search strategy for the Web of science

No.	Search Query	Results
#1	shock, septic'/exp OR 'sepsis'/exp OR 'septic shock':ti,ab OR 'shock, septic':ti,ab OR 'shock, endotoxi*':ti,ab OR 'endotoxin shock*':ti,ab OR 'endotoxic shock*':ti,ab OR 'sepsis':ti,ab OR 'bloodstream infection*':ti,ab OR 'infection, bloodstream':ti,ab OR 'pyemia*':ti,ab OR 'pyohemia*':ti,ab OR 'pyaemia*':ti,ab OR 'septicemia*':ti,ab OR 'poisoning, blood':ti,ab OR 'poisonings, blood':ti,ab OR 'blood poisoning*':ti,ab OR 'severe sepsis':ti,ab OR 'sepsis, severe':ti,ab	411,010

#2	macrolides'/exp OR 'erythromycin'/exp OR 'azithromycin'/exp OR 'clarithromycin'/exp OR 'erythromycin estolate'/exp OR 'erythromycin ethylsuccinate'/exp OR 'ketolides'/exp OR 'roxithromycin'/exp OR 'macrolide*':ti,ab OR 't-stat':ti,ab OR 'erymax':ti,ab OR 'erycette':ti,ab OR 'ilotycin':ti,ab OR 'azythromycin':ti,ab OR 'sumamed':ti,ab OR 'cp-62993':ti,ab OR 'zithromax':ti,ab OR 'azitrocin':ti,ab OR 'azadose':ti,ab OR 'ultreon':ti,ab OR 'zitromax':ti,ab OR 'clarithromycin':ti,ab OR '6-o-methylerythromycin':ti,ab OR 'te-031':ti,ab OR 'a-56268':ti,ab OR 'biaxin':ti,ab OR 'erythromycin estolate':ti,ab OR 'erythromycin*':ti,ab OR 'ilosone':ti,ab OR 'erythromycin ethylsuccinate':ti,ab OR 'wyamycin e':ti,ab OR 'monomycin':ti,ab OR 'e-mycin e':ti,ab OR 'erythroped':ti,ab OR 'ketolides':ti,ab OR 'roxithromycin':ti,ab OR 'rulide':ti,ab OR 'rulid':ti,ab OR 'roxi*':ti,ab OR 'ru-28965':ti,ab OR 'ru-965':ti,ab	406,897
#3	#1 AND #2	17,707
#4	#3 NOT 'case report'/de NOT 'review'/it AND [english]/lim AND [adult]/lim NOT [medline]/lim	545
#5	retracted article	1
	Total	544

Supplementary Table S5 Quality assessment of observational studies using the nine-star Newcastle-Ottawa Scale

Randomized controlled studies	Selection bias		Performance bias	Detection bias	Attrition bias	Reporting bias	Other bias
	Random sequence generation	Allocation concealment	Blinding of partcipants and personnel	Blinding of outcome assessment	Incomplete outcome data	Selective reporting	Other sources of bias
Giamarellos-Bourboulis, 2008	Low	Low	Low	Low	Low	Low	Low
Giamarellos-Bourboulis, 2014	Low	Low	Low	Low	Low	Low	Low
Karakike, 2022	Low	Low	Low	Low	Low	Low	Low

Cohort studies	Selection				Comparability		Outcome			Quality of score
	Representa-tiveness of the exposed cohort	Selection of the non-exposed cohort	Ascertai-nment of exposure	Outcome of interest not present at start of study	Comparab-ility: main	Comparability: others	Assessment of outcome	Long follow-up enough	Adequacy of follow-up	
Adrie, 2013	*	*	*	*	*		*	*	*	8
Afshar, 2016	*	*	*	*	*		*	*	*	8
Behal, 2022		*	*	*	*		*	*	*	7
Johnson, 2023	*	*	*	*	*		*		*	7
Kawamura, 2016		*	*	*	*		*	*	*	7
Reijnders, 2022	*	*	*	*	*		*	*	*	8
Restrepo, 2009	*	*	*	*	*		*	*	*	8
